# Rootstock effects on scion phenotypes in a ‘Chambourcin’ experimental vineyard

**DOI:** 10.1038/s41438-019-0146-2

**Published:** 2019-05-01

**Authors:** Zoë Migicovsky, Zachary N. Harris, Laura L. Klein, Mao Li, Adam McDermaid, Daniel H. Chitwood, Anne Fennell, Laszlo G. Kovacs, Misha Kwasniewski, Jason P. Londo, Qin Ma, Allison J. Miller

**Affiliations:** 1 Department of Plant and Animal Sciences, Faculty of Agriculture, Dalhousie University, Truro, NS B2N 5E3 Canada; 2 Department of Biology, Saint Louis University, 3507 Laclede Avenue, St. Louis, MO 63103-2010 USA; 3 Donald Danforth Plant Science Center, 975 North Warson Road, St. Louis, MO 63132-2918 USA; 4 Department of Math & Statistics, BioSNTR, South Dakota State University, Brookings, SD 57006 USA; 5 Department of Horticulture, Michigan State University, East Lansing, MI 48824 USA; 6 Department of Computational Mathematics, Science and Engineering, Michigan State University, East Lansing, MI 48824 USA; 7 Department of Agronomy, Horticulture & Plant Science, BioSNTR, South Dakota State University, Brookings, SD 57006 USA; 8 Department of Biology, Missouri State University, 901S. National Avenue, Springfield, MO 65897 USA; 9 Department of Food Science, University of Missouri, 221 Eckles Hall, Columbia, MO 65211 USA; 10 United States Department of Agriculture, Agricultural Research Service: Grape Genetics Research Unit, 630 West North Street, Geneva, NY 14456-1371 USA

**Keywords:** Plant sciences, Natural variation in plants, Plant signalling

## Abstract

Understanding how root systems modulate shoot system phenotypes is a fundamental question in plant biology and will be useful in developing resilient agricultural crops. Grafting is a common horticultural practice that joins the roots (rootstock) of one plant to the shoot (scion) of another, providing an excellent method for investigating how these two organ systems affect each other. In this study, we used the French-American hybrid grapevine ‘Chambourcin’ (*Vitis* L.) as a model to explore the rootstock–scion relationship. We examined leaf shape, ion concentrations, and gene expression in ‘Chambourcin’ grown ungrafted as well as grafted to three different rootstocks (‘SO4’, ‘1103P’ and ‘3309C’) across 2 years and three different irrigation treatments. We found that a significant amount of the variation in leaf shape could be explained by the interaction between rootstock and irrigation. For ion concentrations, the primary source of variation identified was the position of a leaf in a shoot, although rootstock and rootstock by irrigation interaction also explained a significant amount of variation for most ions. Lastly, we found rootstock-specific patterns of gene expression in grafted plants when compared to ungrafted vines. Thus, our work reveals the subtle and complex effect of grafting on ‘Chambourcin’ leaf morphology, ionomics, and gene expression.

## Horticulture: dissecting the effects of grafting

Researchers in the US have uncovered how rootstocks affect scions following grafting, which will help to develop more resilient crops. A team led by Allison Miller of Saint Louis University grafted the ‘Chambourcin’ grape variety onto three different rootstocks and investigated the effect on leaf shape, ion concentration, and gene expression in the scion. They discovered that the rootstock influenced how leaf shape changed in response to different irrigation conditions. A similar irrigation-dependent effect of rootstock was found for the ion composition in the shoot. The team also identified roughly 100 genes with altered expression in each of the grafted vines, including five genes which were altered by all three graft combinations. These findings are a first step toward understanding the relationship between rootstocks and scions and modulating it to produce crops better adapted to challenging environments.

## Introduction

Root and shoot systems operate in dramatically different environments and provide unique roles within a plant. These functionally distinct below- and above-ground parts are inextricably linked at the organismal level. Understanding the impact of roots on shoot phenotypes, and conversely, how variation in the shoot influences the roots of a plant, are fundamental questions in plant biology. A further understanding of this interaction also has important agricultural implications, since selection for traits like root architecture and physiology can enhance stress tolerance and yield^1^.

In over 70 major crops, selection for root and shoot system traits have been decoupled through the process of grafting. Grafting is an ancient horticultural technique that creates a composite plant by surgically attaching the roots from one plant (the rootstock) to the shoot (the scion) of another, joining their vascular and cambial systems^2^. Grafting was originally implemented for easier clonal propagation, but today this method achieves a variety of agricultural goals, including drought tolerance, dwarfing, and disease resistance^1^. Beyond its practical implications, grafting offers an unique opportunity to independently manipulate parts of the plant to understand how roots impact shoots, and vice versa.

Grapevine (*Vitis* L.) is an excellent model for examining rootstock–scion interactions due to the ease of cloning, available genomic resources, ability to grow across diverse environments, and high economic value. Widespread grafting of grapevine began in the late 19th century after the European wine industry was devastated by the spread of phylloxera (*Daktulosphaira vitifoliae* Fitch), an aphid-like insect introduced from North America. Unlike many North American *Vitis* species, roots of the European wine grape *Vitis vinifera* L. cannot tolerate phylloxera attacks, which lead to a rapid decline in vigour and often death^3^. However, susceptible *V. vinifera* vines can be grafted to phylloxera-tolerant North American *Vitis* rootstocks, thus circumventing phylloxera sensitivity. Worldwide more than 80% of all vineyards grow vines grafted onto rootstocks composed of American *Vitis* species or hybrids^3^.

Although initial grapevine grafting was driven by the need for phylloxera tolerance, the benefits of grafting have expanded to include resistance to additional pests and pathogens such as nematodes^4^ and increased tolerance to abiotic stresses including drought^5,6^, salinity^7^, and calcareous soils^8^. Lastly, grafting can modify mineral nutrition^9^, scion vigor^10^, rate of ripening^11^, and fruit phenolic compounds^12^. Thus, grafting is a valuable tool for improving grapevine fruit quality and response to stress.

Most commonly used grapevine rootstocks are hybrid derivatives of two or three phylloxera-tolerant native North American species, *Vitis riparia* Michx. and *Vitis rupestris* Scheele, which root easily from dormant cuttings, and *Vitis cinerea* var. *helleri* (L.H. Bailey) M.O. Moore (syn. *Vitis berlandieri*), which is adapted to chalky soils^13^. Despite the global diversity of soils, climates, and grape varieties, only a handful of rootstock cultivars derived from these three species are in widespread use^3^.

Over a century of grafting grapevines has resulted in a wealth of information characterizing graft-transmissible traits. However, many aspects of rootstock and scion interactions are still poorly understood. One area of interest is leaf morphology, which traditionally played a major role in the field of ampelography and was used to distinguish grapevine cultivars^14^. We examined the ability of quantitative measurements of leaf shape to discern subtle effects of rootstocks on scion development. We also examined the effect of rootstocks on leaf ionomic profiles, consisting of mineral nutrients and trace elements^15^. Rootstocks which limit or enhance the transport of particular elements could facilitate grape-growing in regions with suboptimal soil conditions. Lastly, we compared patterns of gene expression in ungrafted and grafted vines. Recent work has described rootstock-induced differential gene expression in response to soil conditions such as nitrogen availability^16^. However, research so far has focused primarily on evaluating rootstocks with known contrasting effects under stressful conditions, and a broader understanding is still needed. Ultimately, understanding how rootstocks can affect scion traits will further our understanding of root–shoot communication and provide insight when selecting parents or progeny in a rootstock breeding program.

To better understand the rootstock–scion relationship, we examined ‘Chambourcin’, a French-American hybrid grape of commercial importance^17^, grown ungrafted as well as grafted onto three different rootstocks (‘SO4’, ‘1103P’, and ‘3309C’) across two years and three different irrigation treatments. Using comprehensive leaf shape analysis, leaf ion concentrations, and patterns of gene expression, we evaluated whether these scion traits are altered by use of rootstocks.

## Results

### Leaf shape assessed using shape descriptors

**Fig. 1:**
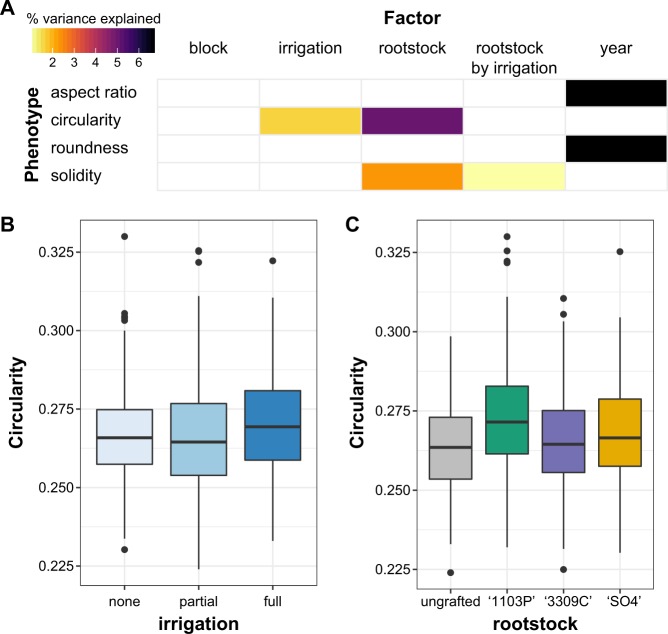
Variation in leaf morphology assessed using the shape descriptors aspect ratio, circularity, roundness and solidity. **a** A linear model was estimated for shape descriptors including the factors block (indicating position in the orchard, as visualized in Fig. S1), year of sample (2014 or 2016), rootstock (ungrafted, ‘1103P’, ‘3309C’, or ‘SO4’), irrigation (none, partial, or full irrigation) and rootstock by irrigation interaction. The percent variance explained by each factor in the model is indicated using color for those factors which explain a significant portion of the variance (*p* < 0.05). **b** Boxplots indicating circularity based on historical irrigation treatment. **c** Boxplots indicating circularity based on rootstock

We examined whether a significant amount of the variation in simple shape descriptors, used to describe leaf morphology, could be explained by block (indicating position in the vineyard, Fig. S1), rootstock (ungrafted, ‘SO4’, ‘1103P’, or ‘3309C’), year of sampling (2014 or 2016), irrigation treatment from the prior year (none, partial, or full) and the interaction between rootstock and irrigation (Fig. 1a, Table S1). The shape descriptors estimated included aspect ratio and roundness. Aspect ratio measures length-to-width ratio, and is calculated by taking the ratio of the major to minor axis of a fitted ellipse around a grapevine leaf. Roundness is the inverse of aspect ratio, and a completely round leaf would have a value of one. We found that a significant amount of variation in aspect ratio (6.64%) and roundness (6.66%) measurements were explained by year of collection.

We also measured variation in leaf morphology using circularity, which is calculated as 4 × (area ÷ (perimeter)^2^. The ratio of area to perimeter squared is sensitive to undulations in the outline of a closed contour, such that grapevine leaves with high levels of serration or lobing have low circularity values, while more entire leaves have higher values. We found variation in circularity was significantly explained by rootstock (5.00%) and irrigation factors (1.66%). We visualized variation in the circularity based on irrigation (Fig. 1b) and rootstock (Fig. 1c), finding that leaves from vines which had full irrigation the year prior tended to have more subtle lobing and serration (i.e., higher circularity values). Circularity values were also higher for leaves of scions grafted to ‘1103P’ rootstocks compared to other rootstock treatments (Fig. 1c).

Lastly, solidity measures area divided by the convex hull area, which is the area as if a rubber band had been placed around the shape (including the area of serrations and lobes). Solidity values close to one describe leaves with little to no lobing, while smaller ratios indicate serrations or lobing. A significant but minor amount of the variance in leaf solidity was explained by rootstock (2.35%) and rootstock by irrigation interaction (1.06%).

### Leaf shape assessed using comprehensive morphometrics

**Fig. 2:**
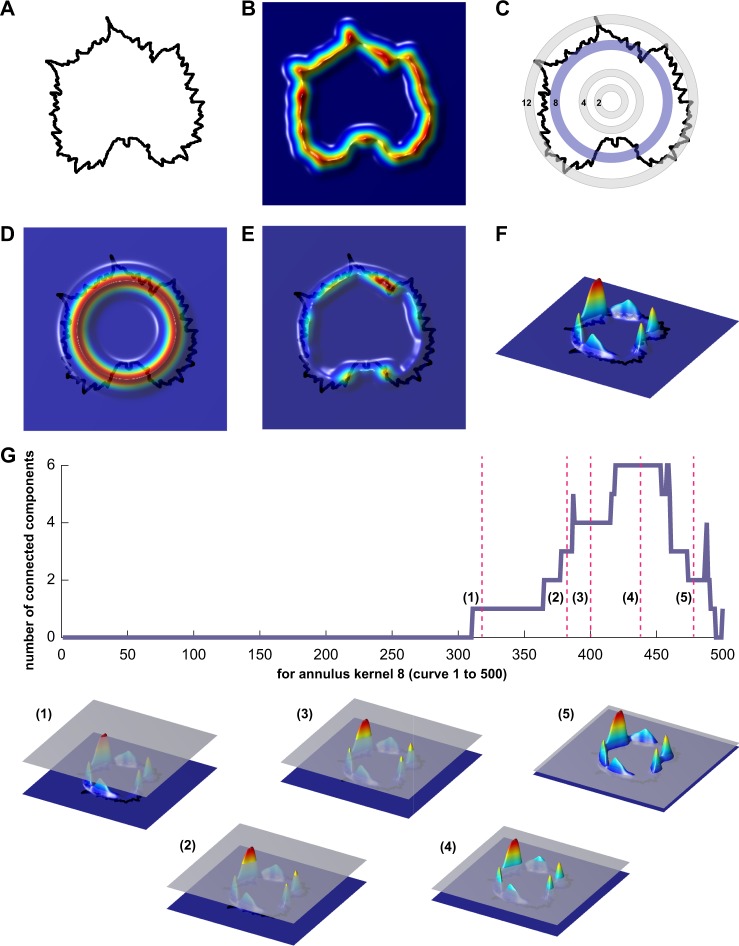
Quantifying leaf shape using persistent homology, a Topological Data Analysis (TDA) method. **a** A 2D point cloud represents each leaf contour. **b** A Gaussian density estimator estimates the density of neighboring pixels around each pixel. Pixels near serrations and lobes tend to have higher density values. **c** 16 concentric rings are used to partition the data as an **d** annulus kernel. **e** Multiplication of the annulus kernel by the Gaussian density estimator isolates sub-features of the leaf. **f** A side projection shows clearly the isolated density features of the leaf. **g** Proceeding from high density values to low (1–5) the number of connected components (a topological feature) is recorded as a function of density. The resulting curves from each ring are discretized and quantify leaf shape

To examine the contours of grapevine leaf shape more comprehensively, we used a persistent homology approach to describe the outline of the leaf (Fig. 2). We applied a Gaussian density estimator to each pixel in the outline, and thus, the pixels with more neighbouring pixels are red, and pixels with few neighbouring pixels are blue (Fig. 2a, b). In the context of leaf shape, pixels in serrations or lobes tend to have more neighbors than pixels that lie on relatively straight edges. We applied the Gaussian density estimator to 16 concentric annuli (rings) emanating from the geometric center (Fig. 2c–f). In Fig. 2g, the values of the Gaussian density filtration function are visualized directly on a grapevine leaf shape. The number of connected components is monitored. As the filtration function is passed through, connected components will arise or merge with each other. This results in a Euler characteristic curve for each of the 16 rings, which were discretized and used as the input for principal components analysis (PCA) (Fig. 2, Table S2).

**Fig. 3:**
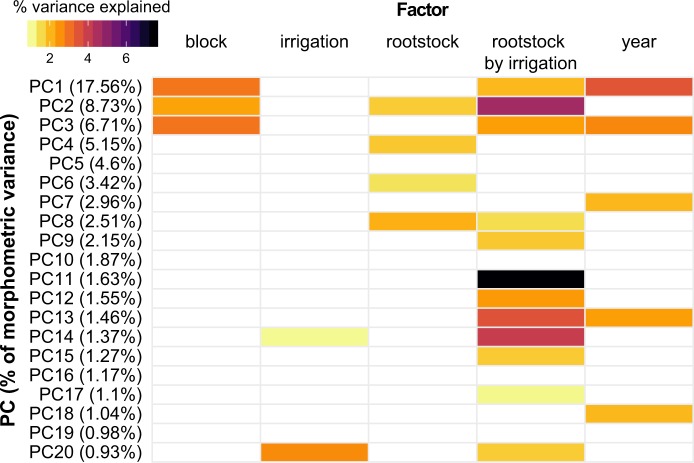
A linear model was estimated for morphometric PCs 1 to 20. The model included block (based on position in the orchard, as indicated in Fig. S1), year (2014 or 2016), rootstock (ungrafted, ‘1103P’, ‘3309C’, or ‘SO4’), irrigation (none, partial, or full), and rootstock by irrigation interaction (Fig. 3). The amount of variance explained by each PC is listed in parenthesis with the first 20 PCs capturing a total of 68.13% of the variance in leaf shape. Only factors which explained a significant portion of the variance (*p* < 0.05) are plotted. The percent variance explained by each factor is indicated using color

For the first principal component (PC1), which explained 17.56% of the total variation in leaf shape captured using this persistent homology based approach, the primary source of variation described by our model was year (3.47%), followed by block (2.90%). However, across many morphometric PCs examined, the rootstock by irrigation interaction described more variation than any other factor assessed. Of the 26 significant relationships (*p* < 0.05) identified for PCs 1 to 20, 12 were for rootstock by irrigation interaction, followed by five for year. In contrast, rootstock explained a significant portion of the variation in leaf shape for four PCs, while irrigation was a significant factor for two PCs. Thus, changes in leaf shape measured using this comprehensive morphometric approach are most affected by the interaction of rootstock by irrigation, although year and block (which reflects position in the vineyard) are important as well.

### Ion concentrations

**Fig. 4:**
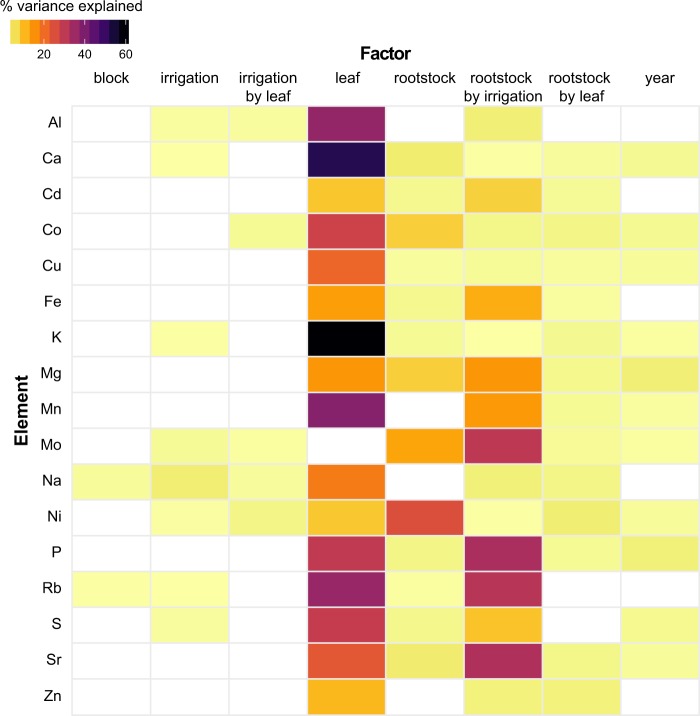
A linear model was estimated for each element measured using ionomics. The model included block (position in the orchard, as indicated in Fig. S1), irrigation (none, partial or full), leaf (old, mid, or young based on position of the leaf in the shoot sampled), irrigation by leaf interaction, rootstock (ungrafted, ‘1103P’, ‘3309C’, or ‘SO4’), rootstock by irrigation interaction, rootstock by leaf interaction, and year of sampling (2014 or 2016). Only factors which explained a significant portion of the variance (*p* < 0.05) are plotted. The percent variance explained by each factor is indicated using color

We used the same linear model approach to estimate which factors described the most variation in the 17 elements we examined for leaf ionomics (Fig. 4, Table S3). In addition to the factors considered for leaf morphology, we assessed leaf position along the shoot (‘leaf’), a reflection of leaf developmental stage. As a result, our model identified potential factors contributing to differences in ion concentrations including block (position in the orchard, as indicated in Fig. S1), irrigation (none, partial or full), leaf (old, mid, or young based on position of the leaf in the shoot sampled), irrigation by leaf interaction, rootstock (ungrafted, ‘1103P’, ‘3309C’, or ‘SO4’), rootstock by irrigation interaction, rootstock by leaf interaction, and year of sampling (2014 or 2016). The concentrations of ions in ‘Chambourcin’ leaves were most affected by leaf position, which explained a significant amount of the variance for 16 of the 17 elements we examined, ranging from 7.85% for nickel (Ni) to 60.89% for potassium (K). Over 50% of the variance in Calcium (Ca) and over 36% of the variance in manganese (Mn), aluminium (Al), and rubidium (Rb) could be explained using leaf position.

In addition to the essential role of shoot position in determining ion concentration, rootstock explained a substantial amount of variation in ion profile: it was a significant factor for 13 elements, most notably Ni, where it explained 24.94% of the variation. Lastly, the interaction between rootstock and irrigation was a significant factor for 17 elements, explaining over 30% of the variance for phosphorus (P), strontium (Sr), Rb, and molybdenum (Mo). In comparison, all other factors explained a maximum of 3.75% of the variation for any particular element.

**Fig. 5:**
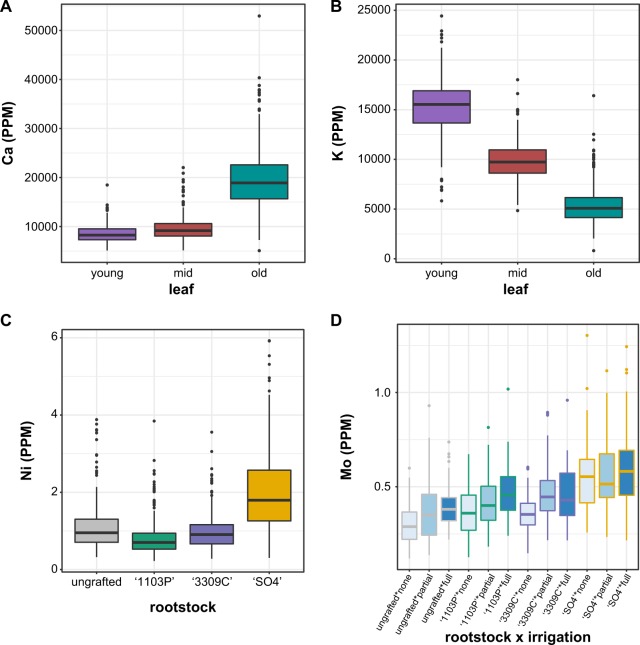
Boxplots showing the distribution of elements by the factor that explained the largest amount of variance. The distribution visualized are: **a** Ca based on leaf position **b** K based on to leaf position **c** Ni based on to rootstock **d** Mo based on rootstock by irrigation interaction

By examining variation for each element across these factors of interest (Fig. S2) we were able to observe several trends (Fig. 5). For example, we found that Ca concentration increased in older ‘Chambourcin’ leaves (Fig. 5a), while K concentration decreased in older leaves (Fig. 5b). Across different rootstock treatments, the leaves of ‘Chambourcin’ grafted to ‘SO4’ generally had the highest concentration of Ni (Fig. 5c). Mo concentrations tended to increase from vines grown ungrafted, to ‘1103P’, to ‘3309C’, to ‘SO4’. Within a particular rootstock, vines which had been fully or partially irrigated the previous season tended to have ‘Chambourcin’ leaves with higher concentrations of Mo than those which had not been irrigated previously (Fig. 5d).

### Gene expression

RNA-sequencing was performed on the first fully open leaf (removing the petiole) from the tip of a shoot from each scion sampled. We used normalized expression data (RPKM) values to test for positively enriched VitisNet Pathways by comparing ‘Chambourcin’ grafted to each individual rootstock with ungrafted ‘Chambourcin’ vines. We identified eight unique enriched pathways for each rootstock, including enrichment for circadian rhythm and phenylalanine metabolism pathways in ‘1103P’. We also combined all grafted ‘Chambourcin’ and compared them to ungrafted vines to determine the impact of grafting, identifying 17 enriched pathways in grafted vines. The full list of pathways identified is available in Table S4.

**Fig. 6:**
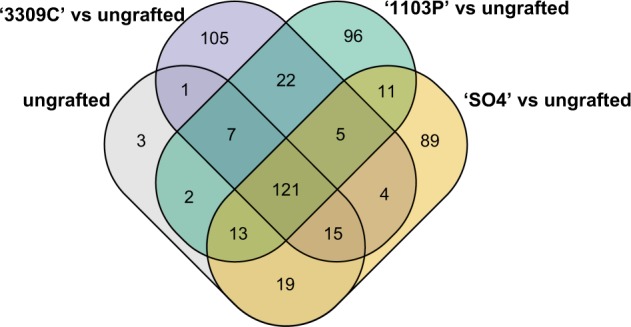
The Venn diagram includes genes with significant temporal expression changes in ungrafted ‘Chambourcin’. Expression profiles of these genes were compared to leaves sampled from ‘Chambourcin’ grafted to each rootstock to determine significant differences between groups

Next, we determined which genes with significant temporal expression changes in leaves sampled from ungrafted ‘Chambourcin’ had differing patterns of expression in ‘Chambourcin’ scions grafted to each rootstock (Fig. 6). In total, there were 513 genes in leaves sampled from ungrafted ‘Chambourcin’ vines with significant temporal expression changes. Of these genes, 121 did not differ in any of the rootstock treatments. There were five genes with different expression profiles in all three grafted vines compared to ungrafted vines. Among these five genes, only one, an isoamylase protein, is annotated. Relative to ungrafted vines, there were 105 genes with significantly different expression patterns only in ‘Chambourcin’ grafted to ‘3309C’, 96 which differed only in ‘Chambourcin’ grafted to ‘1103P’, and 89 which differed only in ‘Chambourcin’ grafted to ‘SO4’ (Table S5; Table S6). Pathway enrichment analysis was used to examine these rootstock specific genes. While no major enrichment was observed for the ‘3309C’ and ‘SO4’ genes, ‘1103P’ vines had a significant number of genes involved in phenylalanine metabolism (four DEGs, *p* = 4.84 × 10^−6^) and auxin biosynthesis (three DEGs, *p* = 1.74 × 10^−5^) pathways (Table S6).

## Discussion

Grafting offers an excellent opportunity to understand how roots modulate scion phenotypes through experimental manipulation. Our study uses grapevine as a model to quantify the effect of rootstock on leaf shape, ion concentrations, and gene expression in the scion.

### Leaf shape is modulated by the interaction of rootstock and irrigation

The grapevine genus is well-known for extensive within and among-species variation in leaf shape^14,18^. Previous work demonstrated that the genetic underpinnings of leaf shape are evolutionarily conserved within species, while developmental constraints and environmental influences such as light, temperature, and water availability affect leaf shape variation among genotypes and within individuals^19–21^. We collected leaves from approximately the same developmental stage (i.e. position on the shoot) from vines of ‘Chambourcin’ to minimize leaf shape differences due to position along the vine (i.e., heteroblasty^22^).

We measured leaf shape using two approaches: shape descriptors, a common digital morphometric technique that captures simple shape differences; and persistent homology, a comprehensive morphometric technique, which allowed us to detect complex and subtle variation in shape. Using these morphometric techniques, we were able to measure variation in shape that would be missed by visual observation alone. Indeed, in all cases the factors we assessed explained less than 8% of the variation for a particular measurement. These subtle and complex changes can only be captured using precise morphometric techniques.

Across the factors we assessed as potentially contributing to variation in leaf shape, interannual variation (year) between leaves sampled in 2014 and leaves sampled in 2016 explained a significant amount of variation for measurements taken using both shape descriptors and persistent homology approaches. However, shape descriptors generally did not vary due to rootstock by irrigation effects. For example, ~1% of variation in the solidity measurement was significantly explained by rootstock by irrigation, while the same interaction effect was a significant factor for 12 of the 20 morphometric PCs examined, explaining up to 7.53% of the variation for a particular PC. Persistent homology uses a comprehensive method for quantifying shape and likely picks up on intricate leaf shape differences that traditional methods miss. Thus, this method allowed us to demonstrate that even a historical irrigation effect from previous years can interact with the roots to shift the shape of ‘Chambourcin’ leaves in measurable ways.

Recent work in apple described a heritable basis for leaf shape^23^, measured using the same persistent homology approach implemented here. Our work suggests that rootstocks could be used to modulate variation in leaf shape in the scion, especially under varying environmental conditions such as access to water. We find further evidence that rootstocks can modulate scion development and patterning, and that signals from the root affect patterning within scion meristems. Although some molecular evidence supports such long-distance coordination of developmental patterning^24^, its prevalence and manifestation across plants remains understudied.

In addition to our work, other studies in grapevine have identified scion leaf shape modulation under different rootstock and irrigation treatments. Tsialtas et al. (2008) examined ‘Cabernet-Sauvignon’ grafted on ‘1103P’ and ‘SO4’ rootstocks under three different irrigation treatments at three time points (bunch closure, veraison, and ripeness). Tsialtas et al. found that while rootstock, irrigation, and rootstock by irrigation did not have a significant effect on leaf morphology, the rootstock by irrigation by time interaction was significant for all leaf shape measurements assessed^25^. In addition, recent work evaluating the leaves of ‘Italia’ grapes grown ungrafted and grafted to two rootstocks under two irrigation conditions, found that leaf area was significantly affected by rootstock by irrigation interaction^26^. Thus, it is clear that the influence of rootstock on leaf shape is a complicated relationship that is at least partially influenced by other factors including irrigation. Future sampling of leaves throughout the growing season for morphometric analysis and gene expression may help identify more precisely the effect of rootstock by irrigation on leaf shape in future studies.

### Ion concentrations in the scion are primarily affected by leaf position, but also influenced by rootstock and rootstock by irrigation interaction effects

The interaction between root system and elemental composition in grapevine shoot systems is an area of great research interest in viticulture^9,27^. The grapevine industry places enormous importance on terroir, the physical environment in which a grapevine is grown, to determine the sensory experience and economic value of wine^28^. Indeed, research shows that available soil nutrients can be transported and stored in different plant tissues^29^. In addition, rootstock selection can affect ion uptake^30^, which can have a pronounced effect on wine quality. Soil elements such as Mg, Mn, and Mo are present in berries throughout wine production (i.e., harvest to bottling), depending on the concentration of these elements in a given geographic region^28^.

Our work identifies the position of the leaf along the shoot (the developmental stage of the leaf) as the largest source of variation observed in most ions. In previous work, position along the main stem had a profound effect on seed composition in soybean, and indicating that position within a plant can effect ion concentrations.^31^ We examined 17 elements and found that for 13 the primary source of variation explaining ion concentration was leaf position. For example, we observed that younger leaves had lower concentrations of Ca than older leaves. This difference in Ca concentration is likely due to the fact that transpiration is low in young leaves, which must rely on transpiration to transport Ca from the xylem^32^. Al and Mn also decreased in younger leaves, while K and Rb increased. These elements provide examples of the changes that occur in elemental composition as leaves develop and age, regardless of rootstock.

While the primary source of variation in ion concentrations was leaf position, a significant amount of variation was also explained by the interaction between rootstock and irrigation for all 17 elements, while rootstock explained a significant amount of variation for 13 elements. Either rootstock or rootstock by irrigation explained >10% of the variation for Fe, Mg, Mn, Mo, Ni, P, Rb, and Sr. Grafting and the choice of rootstock can have a substantial effect on ion accumulation across diverse crops, including grapes. Rootstocks may induce changes in ion concentrations through mechanisms such as changes in root architecture, water uptake, ion transport, and plant hormones^33^. Previous work determined that grafting ‘Négette’ vines onto ‘SO4’ modified scion uptake of minerals, resulting in higher K and lower Ca and Mg concentrations compared to ‘3309C’ and ‘101-14 Mgt’^34^. While we did not detect a similar pattern in the leaves of ‘Chambourcin’ scions, we found that vines grafted to ‘SO4’ had higher concentrations of Ni than vines grown ungrafted or grafted to ‘3309C’ or ‘1103P’. Across the United States, Ni is highest in serpentine soil areas of California^35^. Serpentine soil increases Ni accumulation in grapevine roots, with previous work identifying a significant positive correlation between Ni in the soil and leaves. However, the transfer of Ni from grapevine roots to grapes was low^36^. While further testing in serpentine soil is still required, our work provides evidence that ‘SO4’ may not be an optimal rootstock choice for high Ni soils, since excess Ni may cause toxicity limiting crop production^37^.

We generally did not find a significant effect of irrigation on ion concentrations. However, our samples were collected prior to the start of irrigation treatments in 2014 and 2016, and thus, any response to irrigation would be due to historical conditions and chronic stress, rather than current, acute stress. Future work sampling throughout the growing season, both before and after the initiation of irrigation treatments, will be required to assess how historical and current water conditions influence ion concentrations.

Beyond assessing variation in each element independently, previous work has demonstrated that elements interact with each other^38^. Consequently, it is not surprising that we find so many elements influenced by the same factor. In fact, leaf position, rootstock, and rootstock by irrigation interaction each explain a significant amount of variation in at least 13 of the 17 elements, and this broad effect may indicate interaction between elements. We observed that the position of a leaf within a shoot, the root system of the vine, and the interaction between roots and historical access to water, all have a critical effect in determining ion concentrations, and a further understanding of these complex relationships is still necessary.

### Rootstocks alter scion gene expression

Grafting can result in changes in gene expression in the scion both indirectly, such as due to changes in water stress^39^, and directly, for example, due to long-distance transmission of mobile microRNAs^40^. Rootstock modulation of scion gene expression in stressful conditions has been demonstrated in many major crops, including citrus^41^ and apple^42^. However, further work is needed to understand the consequences that grafting has on gene expression. Grafting to a common scion provides an excellent opportunity to better understand how grafting impacts shoot system phenotypes in plants under normal growing conditions.

We examined the influence of grafting to different rootstocks by using all expressed genes to assess pathway enrichment. We detected enrichment of the circadian rhythm pathway in ‘1103P’ relative to ungrafted vines. Thus, even within the timespan of sampling (~8 h) it is necessary to consider the impact of time on changes in gene expression. Future work is needed to describe whether the impact of sampling time is rootstock-specific.

Next, we assessed the influence of root systems on gene expression in shoot systems by contrasting gene expression over time in ‘Chambourcin’ grafted to three different rootstocks relative to ungrafted vines. We found a similar number of genes (89–105) with different expression profiles, compared to ungrafted vines, for each rootstock treatment. This relatively low number of genes may indicate that variation in the scion transcriptome is predominantly under local genotype (scion) control and not largely influenced by signalling from the rootstock. This may be due to the life history of grapevine, a liana with typically long distances between roots and shoots. Only five genes were consistent in their patterns of differential expression across all rootstocks when compared to ungrafted vines, providing evidence of a rootstock-specific effect on scion gene expression.

We also performed pathway enrichment analysis using the genes (89–105) with different expression profiles in only one rootstock when compared to ungrafted vines. Similar to results observed prior to modeling the gene expression data to include block (time) as a factor, we observed a significant enrichment of differentially expressed genes involved in phenylalanine metabolism for ‘Chambourcin’ grafted to ‘1103P’. However, we also observed enrichment for auxin biosynthesis genes in vines grafted to ‘1103P’. Although our work examined leaf tissue, these results are supported by previous work comparing ‘Cabernet Sauvignon’ grafted to ‘1103P’ and ‘M4’ rootstocks which identified a link between rate of ripening and auxin metabolism, finding that genes involved in auxin action were one of the main categories with a rootstock effect in the berry^43^. Our current work did not examine variation in berries, but indicates that there may be a shared graft-transmissible response in gene expression patterns in the leaves. However, future work is needed in order to measure any direct phenotypic consequence of differential gene expression of auxin biosynthesis genes.

Most work examining rootstock effects on scion gene expression focuses on variation under conditions of stress^44,45^. In comparison, our work examined the effect of multiple rootstocks under neutral environmental conditions, and this difference likely explains the subtle but quantifiable effect of rootstock on scion gene expression described here. We find that the graft-transmissible effects on a common scion are rootstock-specific and that time of sampling may play a significant role in rootstock effects but that further work and phenotyping is still needed to explore these complex interactions.

## Conclusions

Our work provides an initial description of the subtle and complex effect of grafting on leaf morphology, ion concentrations, and gene expression in grapevine scions. We found that specific rootstocks have a distinct effect on many of the phenotypes, often interacting with the environment due to historical water availability. In addition, the position of a leaf within a shoot and the position of a vine within the vineyard strongly influenced phenotypic variation. Further work across multiple years and environments is required in order to determine how the relationship between the rootstock and the scion can best be leveraged for adapting grapevines to a changing climate.

## Materials and methods

### Study design and sampling

A ‘Chambourcin’ experimental vineyard was established in 2009 at The University of Missouri Southwest Center Agricultural Experiment Station in Mount Vernon, Missouri, USA. The vineyard includes ungrafted ‘Chambourcin’ vines as well as ‘Chambourcin’ grafted to three different rootstocks (‘3309C’ - *V. riparia* × *V. rupestris*; ‘1103P’ - *V. berlandieri* × *V. rupestris*; ‘SO4’ - *V. berlandieri* × *V. riparia*). The full factorial experiment with varied rootstock and irrigation regimes contains 288 vines: eight replicates of four root–scion combinations x nine vineyard rows with one of three irrigation treatments. The three irrigation regimes are: full replacement of evapotranspiration losses (ET), 50% replacement of ET, and non-irrigated, each replicated three times (Fig. S1). Additional description of the vineyard design and management is available in Maimaitiyiming et al. 2017^46^. All vines received full irrigation during establishment of the vineyard. Irrigation treatments began in 2013 and were initiated several weeks before veraison. Sampling of leaf tissue for morphometric and ionomic analyses occurred on 18 June 2014 and 14 June 2016, while tissue for gene expression analyses was sampled only on 14 June 2016. In both years, sampling occurred prior to the start of irrigation treatments, and thus, any effect of irrigation we observed was due to treatment from the previous year(s), when the buds/leaves/flower of the study years were formed. Data and code for this manuscript are available in a GitHub repository^47^.

### Leaf shape analyses

For leaf shape analyses, the middle four leaves from a single shoot were collected from each vine. Leaves were flattened, stored in plastic bags in coolers in the field, and transferred to a cold room in the lab. Within a few days of collection leaves were imaged using a Canon DS50000 document scanner. Leaves with margin damage were removed from analysis. The resulting dataset included 277 vines with four leaves and six vines with two leaves in 2014, and 284 vines with four leaves, and two vines with two leaves, in 2016.

Leaf scans were converted to binary (black and white) images in Matlab and then analyzed in ImageJ^48^ using four shape descriptors (aspect ratio, circularity, roundness, and solidity), each of which captures a ratio describing variation in lobing and shape^49^. These measurements were averaged across leaves from each plant. We performed linear modeling using the lm() function in R, accounting for variation in block (which reflects vineyard position), irrigation, rootstock, rootstock by irrigation interaction, and year. The percent variance explained by each factor was calculated using the anova() function, and only those with a significant *p*-value (<0.05) were visualized using the ggplot2 package in R^50^.

We comprehensively measured leaf shape using a persistent homology approach previously described by Li et al.^51,52^. Binary images and persistent homology values are available for download^53^.

Persistent homology values were averaged across leaves for each plant and PCA was performed. The first 20 PCs explained 68.13% of the total variance, and thus only these were included in downstream analyses. These PCs were included in a linear model which accounted for variation in rootstock, irrigation (which reflects historical treatment conditions), rootstock by irrigation interaction, year, and block. We calculated how much of the total variance was explained by each trait, and factors explaining a significant portion of the variance (*p* < 0.05) were visualized using the ggplot2 package in R^50^.

### Leaf ion concentration analyses

To investigate ion concentrations in the leaves, three leaves from different developmental stages were collected from a single shoot from each vine. The first leaf sampled came from the first node at the base of the shoot and was the oldest leaf on the shoot. The second leaf sampled (also used for morphometric analyses) came from the middle of the shoot. The third leaf was sampled at the tip of the shoot.

Each sample was processed for ionomic analysis at the Donald Danforth Plant Science Center (St. Louis, MO), as described in Ziegler et al.^54^, including correction for internal standards and standardization based on sample weight using custom scripts, with one minor modification in the dilution method. Samples were digested in 2.5 mL nitric acid and then diluted to 10 mL with ultrapure water. Instead of a second manual dilution, an ESI prepFAST autodiluter diluted samples an additional 5x inline with ultrapure water. The 2014 samples were analyzed using a Perkin Elmer Elan 6000 DRC-e ICP-MS run in standard mode. The 2016 samples were run with a Perkin Elmer NexION 350D ICP-MS with helium mode enabled. The standard used for normalizing samples in 2014 was rerun in December 2017 and all values from 2016 were adjusted to account for variation between instruments. The elements boron (B), selenium (Se), and arsenic (As) did not measure well in at least one year and were subsequently removed from the analysis for both years, resulting in 17 elements for subsequent analysis.

Within each year, we removed extreme outliers for each element with values <0.25 quantile–interquartile range × 5, or >0.75 quantile + interquartile range × 5. After outlier removal, 703–794 samples per element remained for 2014 and 846 samples for 2016 remained. All samples were included in a linear model accounting for leaf, rootstock, irrigation, block, year, rootstock by irrigation interaction, rootstock by leaf interaction, and irrigation by leaf interaction, using the lm() function in R. Since tissue sampling occurred in June prior to the initiation of irrigation treatments, the effect of irrigation describes historical water conditions. The percent variance explained by each factor was calculated, and only those with a significant *p*-value (<0.05) were visualized.

### Gene expression analyses

We used RNA-seq to assess changes in gene expression in leaves of grafted and ungrafted ‘Chambourcin’ vines. Samples were collected from two rows with no irrigation treatment (rows 13 and 15, Fig. S1) on 14 June 2016. Each row was composed of two blocks of vines, and within each block, we sampled two clonal replicates from each rootstock-scion combination, for a total of 32 samples. Samples were collected from row 15 column A to column H, and then from row 13 column A to column H. For each vine, we collected the first leaf at the tip of the shoot that was fully open (~16 mm in diameter). Leaf tissue was immediately flash frozen in liquid nitrogen and transported on dry ice before transferring to a −80 °C freezer for storage.

Total RNA was extracted at the US Department of Agriculture Grape Genetics Research Unit (Geneva, NY) using standard extraction protocols for the Sigma Spectrum Plant RNA kit (Sigma Aldrich, Inc. St. Louis MO) with the following modification: addition of 3% w/v PVP40 added to the lysis buffer. Library construction was performed by Cofactor Genomics (http://cofactorgenomics.com, St. Louis, MO). Total RNA was incubated with mRNA capture beads in order to remove contaminating ribosomal RNA from the sample. The resulting poly(A)-captured mRNA was fragmented. First-strand cDNA synthesis was performed using reverse transcriptase and random primers in the presence of Actinomycin D, followed by second-strand cDNA synthesis with DNA polymerase I and RNase H. Double-stranded cDNA was end-repaired and A-tailed for subsequent adaptor ligation. Indexed adaptors were ligated to the A-tailed cDNA. Enrichment by PCR was performed to generate the final cDNA sequencing library. Libraries were sequenced as single-end 75 base pair reads on an Illumina NextSeq500 following the manufacturer’s protocols. The RNA-sequencing data have been uploaded to the NCBI Sequence Read Archive under BioProject PRJNA507625: SRA Accessions SRR8263050 - SRR8263077.

All samples were quality checked using FastQC v0.11.3(Andrews 2015). Reads were aligned to the 12Xv2 reference genome and the VCost.v3(Canaguier et al. 2017) reference annotation using HISAT2 v2.1.0(Kim et al. 2015). Counts were derived from the alignment with HTSeq^55^. Differential gene expression analysis was performed using the R package DESeq2^56^. After determining differential expression, the raw read counts were normalized using the DESeq2 normalization method of dividing each count by the size factors.

As an initial survey of the potential impact of rootstocks on gene expression, we conducted a Gene Set Enrichment Analysis (GSEA) using GSEA-P 2.0 (http://www.broad.mit.edu/GSEA) and 203 VitisNet pathways including at least 10 genes^57–61^. Enrichment was tested using normalized expression data (RPKM) for all genes, for each rootstock. The gene expression from leaf tissue (‘Chambourcin’ scion) for each rootstock was compared separately to ungrafted ‘Chambourcin’ leaf gene expression, as well as combining all grafted ‘Chambourcin’ and comparing gene expression to ungrafted leaves. For each comparison, we determined which pathways were up-regulated in grafted vines using GSEA. The GSEA-P 2.0 default parameters of 1000 permutations, nominal p-value (*p* < 0.05) and false discovery rate (FDR) *q*-value (*q* < 0.25) were used to identify positive significantly enriched molecular pathways^58^.

Samples from each block were collected chronologically, and thus, each block represented spatial variation as well as a particular time point. We used the maSigPro R package^62,63^ to identify genes with different expression profiles over time when comparing each rootstock to ungrafted vines. To identify genes with significant temporal changes, we performed a regression fit for each gene accounting for all variables (block, replicate, and rootstock) using the p.vector() function with a false discovery rate procedure and 0.05 threshold. In order to compare between groups, we first used the T.fit() function to perform stepwise regression and determine significant variables for each gene. Next, we used the get.siggenes() function with the ‘vars = “groups”’ option, so that ungrafted vines were considered the reference group. This function generated a list of genes with significant temporal expression changes in ungrafted vines and compared expression profiles for each gene between leaves sampled from ungrafted vines and leaves sampled from scions grafted to each rootstock. These results were displayed using the suma2Venn() function.

Lastly, we queried genes identified by maSigPro as having different expression profiles in grafted ‘Chambourcin’ relative to ungrafted ‘Chambourcin’ for statistical enrichment of metabolic and regulatory pathways, to determine if rootstock impacted specific aspects of vine biology. We tested genes for pathway enrichment using the Vitisnet database^60^ and the VitisPathways tool^64^ using 100 permutations, a Fisher’s exact test of *p* < 0.05 and a permuted *p* value of *p* < 0.05.

## Supplementary information

Figure S1. Schematic representation of ‘Chambourcin’ experimental vineyard located at The University of Missouri Southwest Center Agricultural Experiment Station in Mount Vernon, Missouri, USA.

Figure S2. Complete ionomic results for 2014 and 2016 divided based on (A) rootstock (B) leaf position (C) rootstock by irrigation.

Table S1. Results for all factors explaining a significant portion of the variance (*p* < 0.05) for simple leaf shape descriptors consisting of aspect ratio, circularity, roundness and solidity. For each descriptor, the percent variance explained by the factor and the *p*-value are reported.

Table S2.Results for all factors explaining a significant portion of the variance (*p* < 0.05) for morphometric PC1 to 20. For each significant factor for a PC, the *p*-value, percent variance explained by the factor, and percent variance captured by the PC are all reported.

Table S3. Results for all factors explaining a significant portion of the variance (*p* < 0.05) for each element. For each significant factor for an element, the *p*-value and percent variance explained by the factor are reported.

Table S4. VitisNet Pathways that were uniquely positively enriched in a rootstock, or positively enriched in common for all three rootstocks, relative to own-rooted vines. A false discovery rate of 0.25 and nominal *p*-value of 0.05 were used to identify positive enrichment in each rootstock treatment.

Table S5. All genes which were significantly expressed in own-rooted vines were compared to genes in vines grafted to each rootstock to determine which ones were significantly differentially expressed. The results of these comparisons are listed. Annotations are from the VCost.v3 (Canaguier et al. 2017) reference annotation.

Table S6. Genes found to be significantly differentially expressed in vines grafted to only one rootstock when compared to own-rooted vines, or across vines grafted to all rootstocks compared to own-rooted vines, or not differentially expressed across any rootstock treatment, where tested for pathway enrichment.

Supplementary information

## Data Availability

Binary images and persistent homology values are available for download^53^. The RNA-sequencing data have been uploaded to the NCBI Sequence Read Archive under BioProject PRJNA507625: SRA Accessions SRR8263050 - SRR8263077. Data and code for this manuscript are available in a GitHub repository^47^.
