# Supplementary figures and images for: Rootstock effects on scion phenotypes in a ‘Chambourcin’ experimental vineyard
Source: Hortic Res. 2019 May 1;6:64. doi: 10.1038/s41438-019-0146-2 (PMC6491602; doi:10.1038/s41438-019-0146-2)

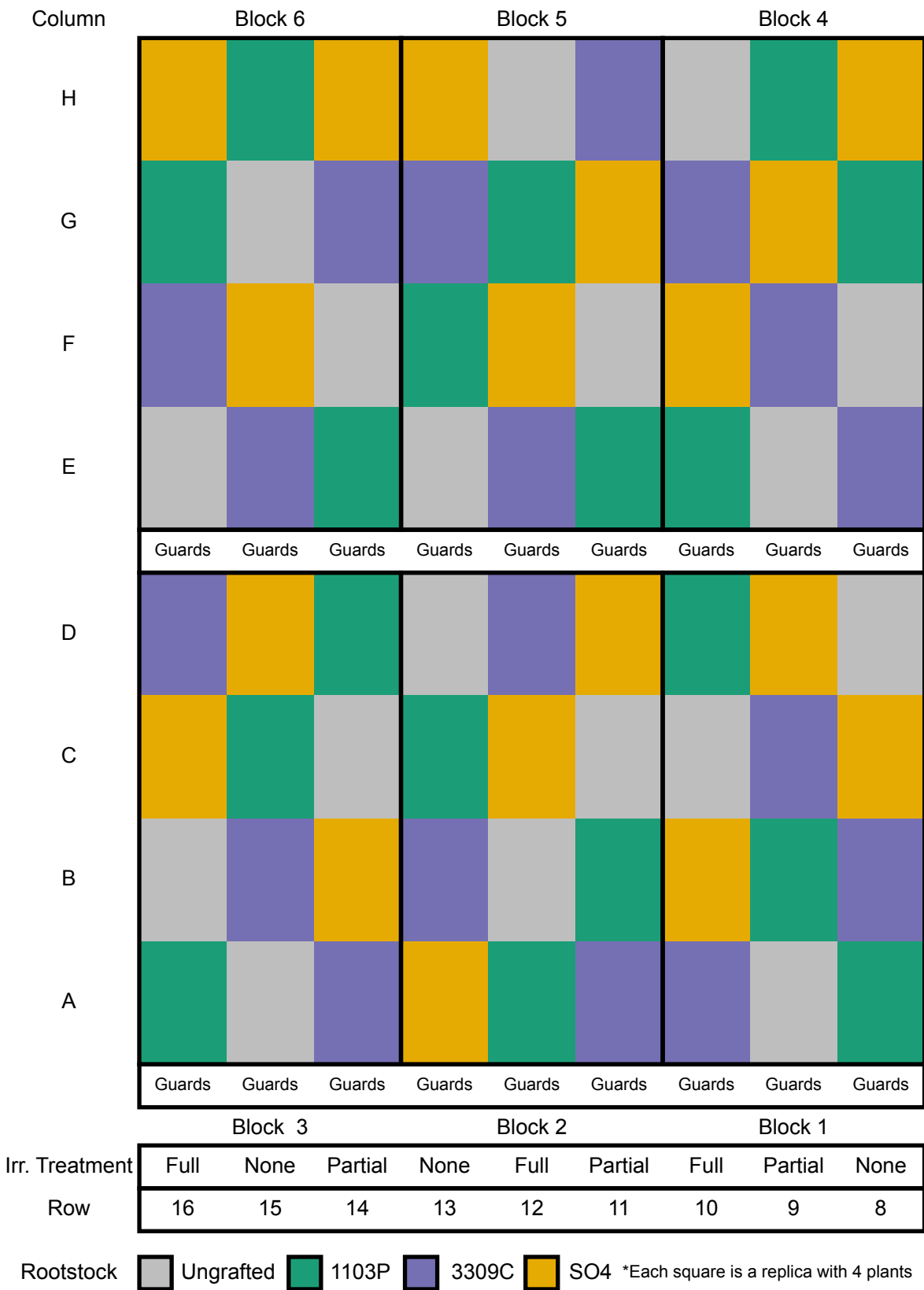

Supplement: Supplementary file 1 — Figure S1. Schematic representation of ‘Chambourcin’ experimental vineyard located at The University of Missouri Southwest Center Agricultural Experiment Station in Mount Vernon, Missouri, USA. [file 41438_2019_146_MOESM1_ESM.pdf]

**A** rootstock

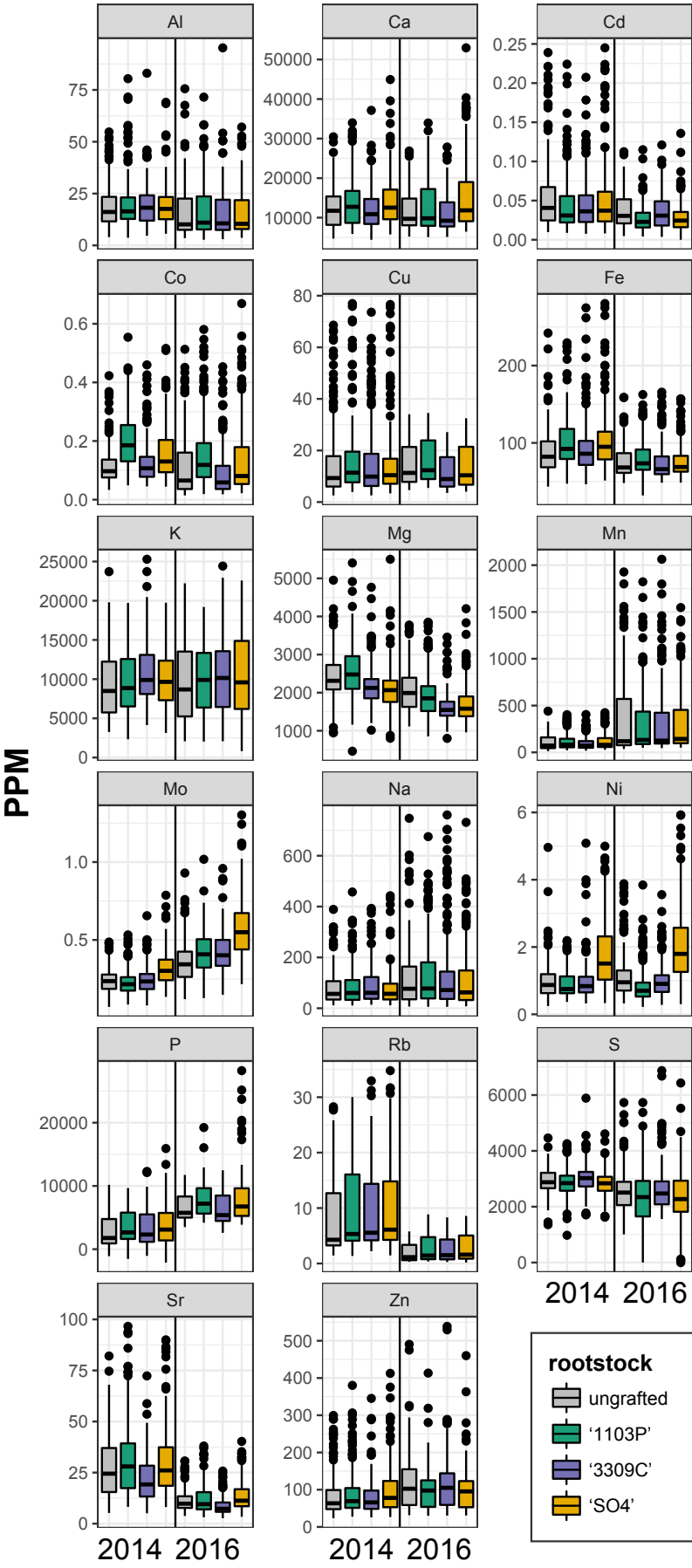

**B** leaf

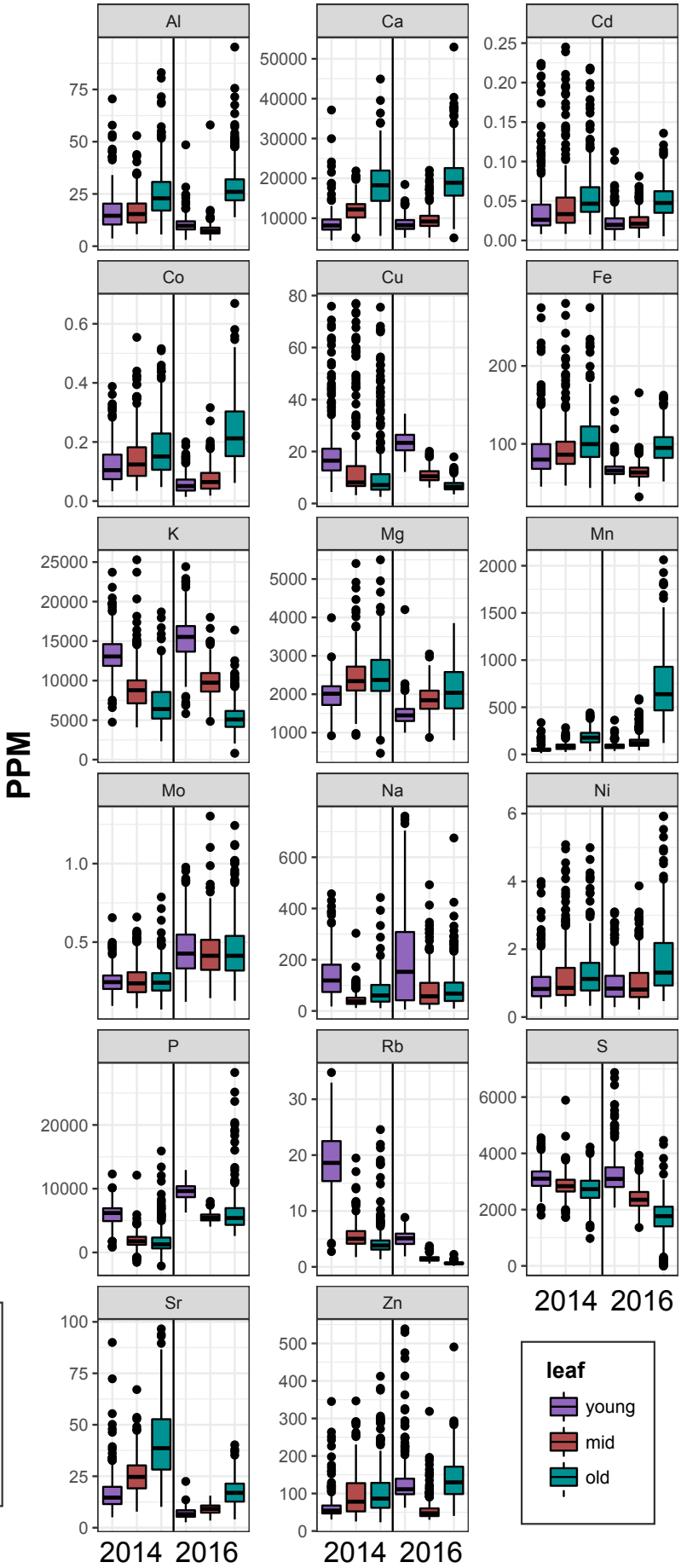

C rootstock by irrigation

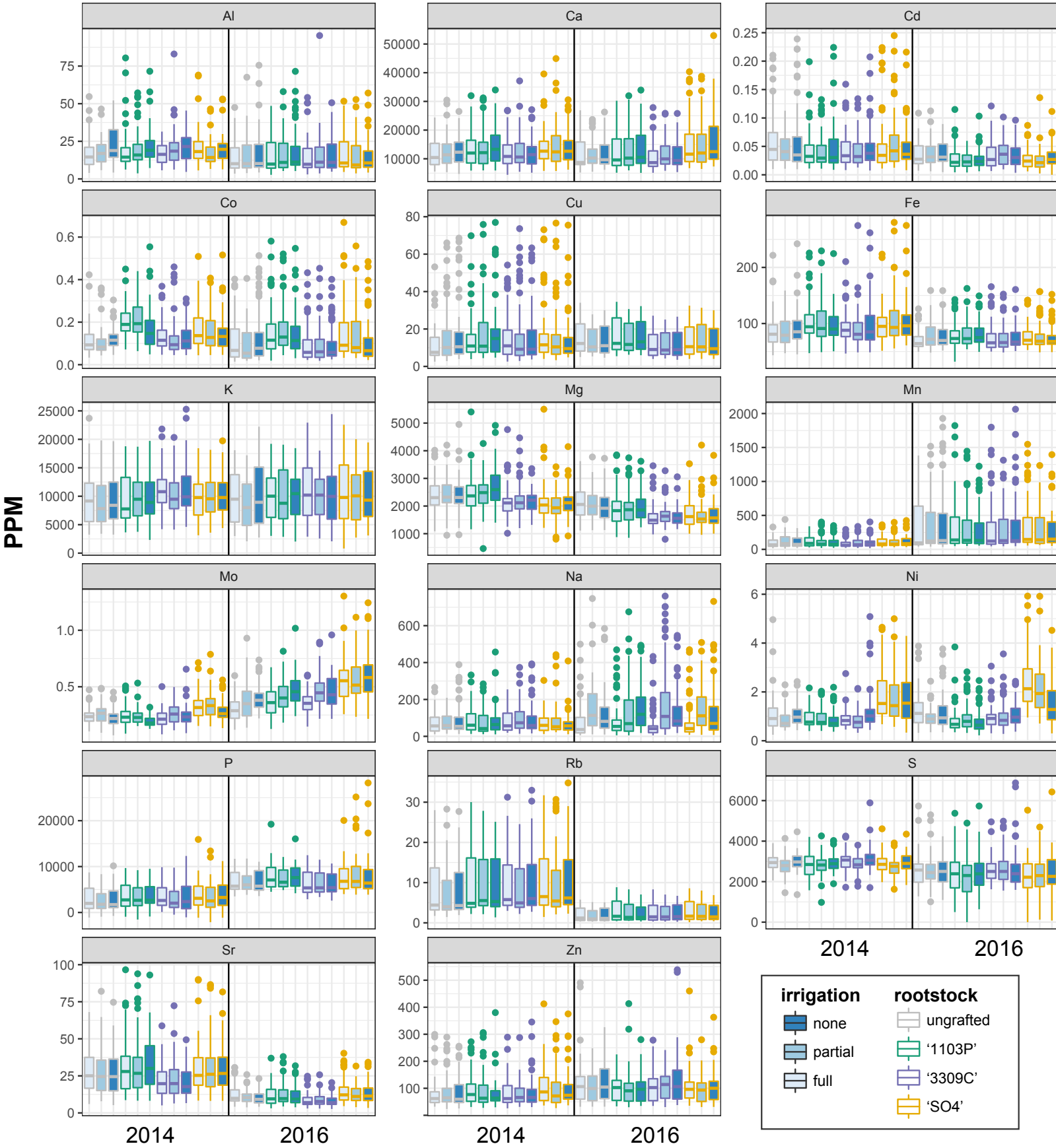

Supplement: Supplementary file 2 — Figure S2. Complete ionomic results for 2014 and 2016 divided based on (A) rootstock (B) leaf position (C) rootstock by irrigation. [file 41438_2019_146_MOESM2_ESM.pdf]
