# Supplementary material for: Rootstock effects on scion phenotypes in a ‘Chambourcin’ experimental vineyard
Source: Hortic Res. 2019 May 1;6:64. doi: 10.1038/s41438-019-0146-2 (PMC6491602; doi:10.1038/s41438-019-0146-2)
Supplement: Supplementary file 9 — Supplementary information [file 41438_2019_146_MOESM9_ESM.docx]

## Supplementary information

**Figure S1**. Schematic representation of ‘Chambourcin’ experimental vineyard located at The University of Missouri Southwest Center Agricultural Experiment Station in Mount Vernon, Missouri, USA.

**Figure S2.** Complete ionomic results for 2014 and 2016 divided based on (A) rootstock (B) leaf position (C) rootstock by irrigation.

**Table S1**. Results for all factors explaining a significant portion of the variance (p < 0.05) for simple leaf shape descriptors consisting of aspect ratio, circularity, roundness and solidity. For each descriptor, the percent variance explained by the factor and the p-value are reported.

**Table S2**. Results for all factors explaining a significant portion of the variance (p < 0.05) for morphometric PC1 to 20. For each significant factor for a PC, the p-value, percent variance explained by the factor, and percent variance captured by the PC are all reported.

**Table S3**. Results for all factors explaining a significant portion of the variance (p < 0.05) for each element. For each significant factor for an element, the p-value and percent variance explained by the factor are reported.

**Table S4**. VitisNet Pathways that were uniquely positively enriched in a rootstock, or positively enriched in common for all three rootstocks, relative to ungrafted vines. A false discovery rate of 0.25 and nominal p-value of 0.05 were used to identify positive enrichment in each rootstock treatment.

**Table S5**. All genes which were significantly expressed in ungrafted vines were compared to genes in vines grafted to each rootstock to determine which ones were significantly differentially expressed. The results of these comparisons are listed. Annotations are from the VCost.v3 (Canaguier et al. 2017) reference annotation.

**Table S6**. Pathway enrichment results for genes found to be significantly differentially expressed in vines grafted to only one rootstock when compared to ungrafted vines, genes which were significant across all rootstocks compared to ungrafted vines, and genes which were not differentially expressed in any rootstock treatment compared to ungrafted vines.
